# Supplementary material for: DNA affects the phenotype of fuel-dependent coacervate droplets
Source: Nat Commun. 2026 Mar 26;17:2953. doi: 10.1038/s41467-026-71024-8 (PMC13031873; doi:10.1038/s41467-026-71024-8)
Supplement: Supplementary file 1 — Supplementary Information [file 41467_2026_71024_MOESM1_ESM.pdf]

Supplementary Information for:

DNA affects the phenotype of fuel-dependent coacervate droplets

Corbin Machatzke,* Anna-Lena Holtmannspötter,* Hannes Mutschler, Job Boekhoven

Affiliations
[1] TU Dortmund University, Otto-Hahn-Strasse 4a, 44227 Dortmund, Germany
[2] Department of Bioscience, School of Natural Sciences, Technical University of Munich, Lichtenbergstrasse 4, 85748 Garching, Germany
* Equal contribution

Supplementary Tables

Supplementary Table 1. Two-sided Welch's t-test for independent samples: p values, t values and degrees of freedom for the maximum turbidity in the presence of different 30mer libraries

**P values**	**Control**	**Unbiased**	**T-rich**	**C-rich**	**G-rich**	**A-rich**
Control	x	0.31	0.019	0.018	0.57	0.01
T values	Control	n30	t	c	g	A-rich
Control	x	-1.25	-4.35	-4.44	-0.66	-4.78
Degrees of freedom	Control	Unbiased	T-rich	C-rich	G-rich	A-rich
Control	x	2.77	3.24	3.21	2.15	3.61

Supplementary Table 2. Two-sided Welch's t-test for independent samples: p values, t values and degrees of freedom for the beginning of droplet dissolution for different 30mer libraries

**P values**	**Control**	**Unbiased**	**T-rich**	**C-rich**	**G-rich**	**A-rich**	**A30**
**Control**	x	0.23	0.52	0.37	0.47	0.10	0.0051
**n30**	0.23	x	0.52	0.64	0.16	0.58	0.003
**A-rich**	0.10	0.58	0.23	0.27	0.13	x	0.0009
**T values**	Control	Unbiased	T-rich	C-rich	G-rich	A-rich	A30
**Control**	x	-1.41	-0.71	-1.00	0.82	-2.53	-14
**n30**	1.41	x	0.71	0.5	1.81	-0.63	-12
**A-rich**	2.53	0.63	1.58	1.41	2.41	X	-34
**Degrees of freedom**	Control	n30	t	C	g	A-rich	A30
**Control**	x	4.00	4.00	3.94	3.17	2.44	2.0
**n30**	1.41	x	4.00	3.94	3.17	2.44	2.0
**A-rich**	2.44	2.44	2.44	2.56	2.14	x	2.0

Supplementary Table 3. Two-sided Welch's t-test for independent samples: p values, t values and degrees of freedom for droplet lifetime for different 30mer libraries.

**P values**	**Control**	**n30**	**T**	**C**	**G**	**A-rich**	**A30**
**Control**	x	0.17	0.71	0.67	0.37	0.47	0.0037
**n30**	0.17	X	0.3	0.19	0.24	0.5	0.00001
**A-rich**	0.47	0.5	0.71	0.68	0.29	x	0.0052
**T values**	Control	n30	T	c	g	A-rich	A30
**Control**	x	-1.94	-0.40	-0.46	1.11	-0.80	-12.20
**n30**	1.94	X	1.34	1.77	1.64	0.80	-26.2
**A-rich**	0.80	-0.80	0.39	0.45	1.38	x	-10.69
**Degrees of freedom**	Control	n30	t	c	g	A-rich	A30
**Control**	x	2.33	3.99	3.74	2.24	3.99	2.33
**n30**	2.33	X	2.31	2.56	2.02	2.31	4.00
**A-rich**	3.99	2.31	4.00	3.67	2.26	x	2.31

Supplementary Table 4. Two-sided Welch's t-test for independent samples: p values, t values and degrees of freedom for droplet lifetimes for different A-rich libraries

**P values**	**A30**	**A5N20A5**	**A10N10A10**	**N10A10N10**	**(A5N5)3**	**A7N16A7**	**N5A20N5**	**A10N20**
**Control**	0.0002	0.10	0.001	0.10	0.57	0.003	0.0007	0.0016

T values	A30	A5N20A5	A10N10A10	N10A10N10	(A5N5)3	A7N16A7	N5A20N5	A10N20
Control	-19.0	2.21	-11.0	-2.21	0.63	-8.00	-13.0	-10.0
Deg. of freedom	A30	A5N20A5	A10N10A10	N10A10N10	(A5N5)3	A7N16A7	N5A20N5	A10N20
Control	3.20	3.45	3.20	3.45	3.45	3.20	3.20	3.20

Supplementary Table 5. Two-sided Welch's t-test for independent samples: p values, t values and degrees of freedom for droplet lifetimes for different G-rich libraries against N30.

P values	G8N14G8	G10A20
N30	0.0001	0.30
T values	G8N14G8	G10A20
N30	-103	1.34
Deg. of freedom	G8N14G8	G10A20
N30	2.00	2.21

Supplementary Table 6. Two-sided Welch's t-test for independent samples: p values, t values and degrees of freedom for critical fuel concentration for different G-rich libraries against no DNA control.

P values	G8N14G8	G10A20
Control	0.52	0.07
T values	G8N14G8	G10A20
Control	0.71	-3.16
Deg. of freedom	G8N14G8	G10A20
Control	4.00	2.44

Supplementary Table 7. Characterization of DNA libraries via HPLC.

Name	retention time [min] @260nm	calibration value (mM/mAu)
N_{30}	5.71	1.50
A_{30}	10.01	1.19
$G_8N_{14}G_8$	5.20	1.07
A-rich	5.82	1.12
G-rich	5.16	1.39
T-rich	6.42	1.34
C-rich	5.17	1.88

Supplementary Table 8. Characterization of peptides.

Name	Amino acid sequence	Mass calculated [g/mol]	Mass observed [g/mol]	Retention time* [min] at 220nm
Peptide	Ac-F(RG)$_3$D-OH	Mw = 962.05	$[MwH]^+$ 963.02<br>$[H_2Mw]^{2+}$ 482.12<br>$[H_3Mw]^{3+}$ 321.78	4.2
Peptide model	Ac-F(RG)$_3$N-NH$_2$	Mw=960.08	$[HMw]^+$ 960.57<br>$[H_2Mw]^{2+\cdot}$ 481.19<br>$[H_3Mw]^{3+}$ 321.20	4.2

*Gradient conditions: 6 min gradient 5%-98% ACN, total runtime 14 min

Supplementary Table 9. List of all motifs enriched in Droplets compared to Supernatant. Sequence corresponds to the consensus sequence of the motif; $Logo_{Info}$ and $Logo_{Prob}$ are the motif logos with information content or nucleotide content as the y-axis; $Position_{Start}$ is the probability that a logo starts at that position; e-value is described in methods above.

Number	Sequence	$Logo_{Info}$	$Logo_{Prob}$	$Position_{Start}$	e-value
1	GGGGGGKGD	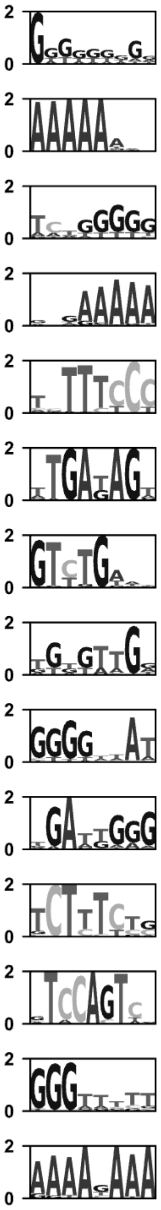	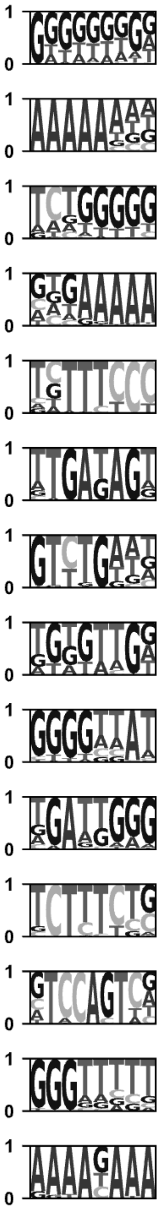	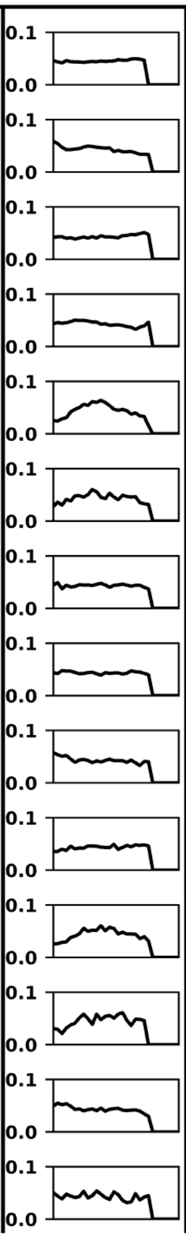	4.5e-320
2	AAAAAAWN				4.0e-096
3	TCTGGGGG				6.1e-029
4	GNGAAAAA				4.5e-016
5	TSTTTCCC				2.8e-010
6	TTGAGAGT				2.7e-009
7	GTYTGAWD				3.4e-009
8	TGKGTTGC				6.9e-009
9	GGGGWTAT				5.7e-008
10	TGATKGGG				2.3e-006
11	TCTTTCTS				7.2e-006
12	GTCCAGTCD				4.4e-005
13	GGGTTTTT				1-1e-002
14	AAAAKAAA				2.5e-002

Supplementary Table 10. All nucleic acid sequences used in this study.

**Name**	**Sequence (5'-3')**
**N_{30}**	(N:0.25A, 0.25G, 0.25T, 0.25C)<br>NNNNNNNNNNNNNNNNNNNNNNNNNNNNNN
**A-rich**	(N:0.76A, 0.08G, 0.08T, 0.08C)<br>NNNNNNNNNNNNNNNNNNNNNNNNNNNNNN
**G-rich**	(N:0.08A, 0.76G, 0.08T, 0.08C)<br>NNNNNNNNNNNNNNNNNNNNNNNNNNNNNN
**T-rich**	(N:0.08A, 0.08G, 0.76T, 0.08C)<br>NNNNNNNNNNNNNNNNNNNNNNNNNNNNNN
**C-rich**	(N:0.08A, 0.08G, 0.08T, 0.76C)<br>NNNNNNNNNNNNNNNNNNNNNNNNNNNNNN
**A_{30}**	AAAAAAAAAAAAAAAAAAAAAAAAAAAAAA
**$N_5A_{20}N_5$**	(N:0.25A, 0.25G, 0.25T, 0.25C)<br>NNNNNAAAAAAAAAAAAAAAAAAAANNNNN
**$N_{10}A_{10}N_{10}$**	(N:0.25A, 0.25G, 0.25T, 0.25C)<br>NNNNNNNNNNAAAAAAAAAANNNNNNNNNN
**$A_5N_{20}A_5$**	(N:0.25A, 0.25G, 0.25T, 0.25C)<br>AAAAANNNNNNNNNNNNNNNNNNNNAAAAA
**$A_7N_{16}A_7$**	(N:0.25A, 0.25G, 0.25T, 0.25C)<br>AAAAAAANNNNNNNNNNNNNNNNAAAAAAA
**$A_{10}N_{10}A_{10}$**	(N:0.25A, 0.25G, 0.25T, 0.25C)<br>AAAAAAAAAANNNNNNNNNNAAAAAAAAAA
**$A_{10}N_{20}$**	(N:0.25A, 0.25G, 0.25T, 0.25C)<br>AAAAAAAAAANNNNNNNNNNNNNNNNNNNN
**$(A_5N_5)_3$**	(N:0.25A, 0.25G, 0.25T, 0.25C)<br>AAAAANNNNNAAAAANNNNNAAAAANNNNN
**$G_8N_{14}G_8$**	(N:0.25A, 0.25G, 0.25T, 0.25C)<br>GGGGGGGGNNNNNNNNNNNNNNGGGGGGGG
**$G_{10}A_{20}$**	GGGGGGGGGGAAAAAAAAAAAAAAAAAAAA
**Unb94**	CGTCCATAGCGCAAATCNNNNNNNNNNNNNNNNNNNNNNNNNN<br>NNNNNNNNNNNNNNNNNNNNNNNNNNNNNNNNNNCTTCACTGG<br>GCTTGTCA

Supplementary Table 11. Diffusion coefficients and t ½ of measured FRAP recoveries in this study. Measurements have been performed in triplicate and the diffusion coefficients are the averages from these triplicates.

**DNA sequence**	**$D_{peptide}$**	**$t_{1/2,peptide}$**	**D_{poly-U}**	**$t_{1/2,poly-U}$**
**N_{30}**	3.3 (+/- 0.27)	0.43 (+/- 0.05)	0.024 (+/-0.003)	28 (+/- 8)
**A_{30}**	3.16 (+/- 0.12)	0.47 (+/- 0.05)	0.006 (+/- 0.0005)	146 (+/- 6)
**no DNA**	3.16 (+/- 0.20)	0.47(+/- 0.05)	0.033 (+/- 0.009)	21 (+/- 7)
**$G_{10}A_{20}$**	1.44 (+/- 0.47)	1.13 (+/- 0.51)	0.002 (+/- 0.0008)	491 (+/- 63)

**$G_8N_{14}G_8$**	1.21(+/- 0.34)	1.21 (+/- 0.34)	0.028 (+/-0.003)	33 (+/- 11)

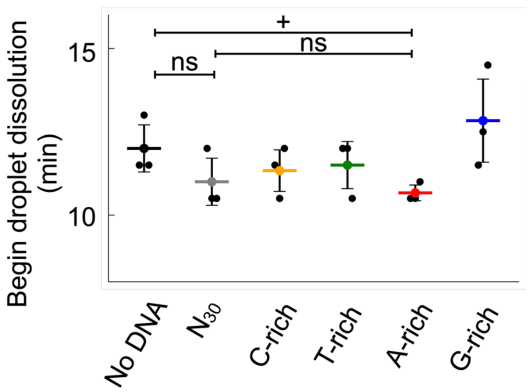

Fig. S1. Longevity of droplets in the presence of different biased randomer libraries. *Droplets were prepared according to standard active droplet sample preparation with 50 µM of DNA. Turbidity was observed as Absorbance at 600 nm on the UV VIS spectrometer for thirty minutes. For droplet longevity, we compare the time point at which the measured turbidity drops below 0.2 as the beginning of the droplet dissolution. Error bars represent the standard deviation from the average of 3 measurements (N=3).* Significance was determined with a two sided Welch's t-test with the no-DNA sample as the control group. P-values are available in Supplementary table 2. Source data is available in the source data file.

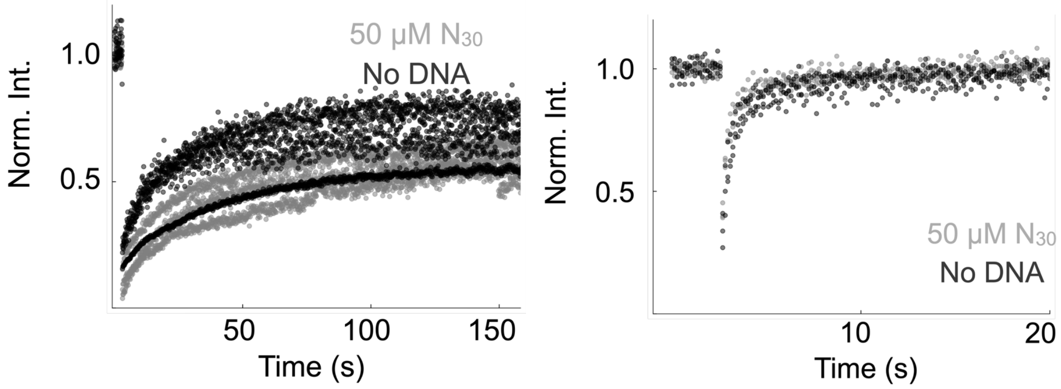

Fig. S2. Comparison of the precursor peptide and poly-U diffusivities with and without 50 µM randomer DNA. *Fluorescence recovery after photobleaching of fluorescently tagged precursor peptide NBD-G(RG)$_3$D-OH,(**A**) used at 1 mM concentration and poly-U (**B**) annealed with 500nM Cy5-A$_{15}$ RNA. Intensity is normalized to an averaged pre-bleach value (Norm. Int.) . Sample conditions were 20 mM peptide, 3 mM peptide model, 5.6 mM poly-U (no DNA Control) or 4.1 mM poly-U with 50 µM N30 (1.5 mM extra charge). The photobleached peptide was observed for 70 s, and the poly-U for 180 s. Measurements were performed in triplicate (three independent experiments (N=3)), diffusion coefficients can be found in Supplementary table 11. Source data is available in the source data file.*

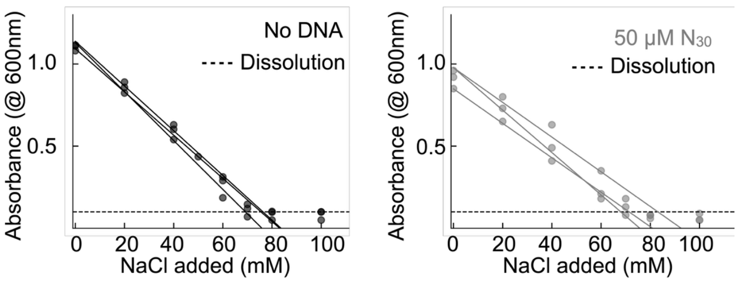

	Control	***N30***
CSC rep.1	*75.5*	*70.0*
CSC rep.2	*69.2*	*71.4*
CSC rep.3	*76.3*	*83.1*
CSC average	*73.7*	*74.1*
CSC standard deviation	*3.91*	*7.91*

Fig. S3. Critical salt concentration in the presence of N_{30} DNA. *Static coacervate samples were titrated according to the standard static droplet protocol with and without 50 µM of random DNA library. 20 µL samples were titrated with concentrated NaCl solution, and the absorbance at 600 nm was measured on the UV VIS spectrometer after salt addition and mixing. The titrations were performed in triplicate (N=3) and each replicate was fit linearly with all datapoints above 0.12, as datapoints below no longer followed the linear trend during dissolution. The critical salt concentrations were determined at the intersection of the extrapolated fits with y=0.1. Source data is available in the source data file.*

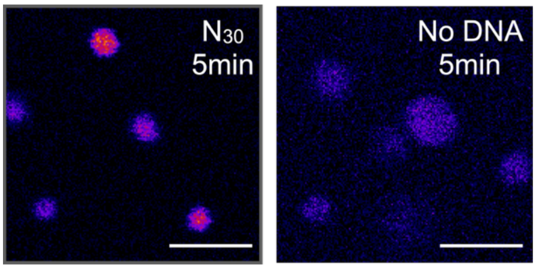

Fig. S4. Droplet morphology in the presence of N30. *Droplets were prepared according to standard conditions with 500 nM Sulforhodamine and observed under the confocal microscope using an IBIDI chamber coated with a 5 %PVA solution. Images were taken after five minutes, 20 µm above the glass. Representative micrograph images were chosen at 5 minutes. Three biological replicates were prepared for each condition (N=3).*

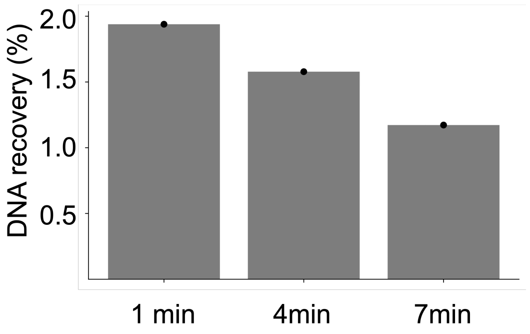

Fig. S5. DNA recovery from active droplets at different timepoints in the cycle. *Droplets were prepared with 23 mM peptide, 4.1 mM poly U 30 mM EDC and 5 µM of a random library Unb94 (94nt). Droplets were spun down for 2 minutes starting at 1, 4 or 7 minutes, separated from the supernatant and DNA was recovered using the same method as for the sequencing library preparation. DNA recovery was determined relative to the input DNA and*

measured via the absorption at 260nm with a Nanodrop instrument. Source data is available in the source data file.

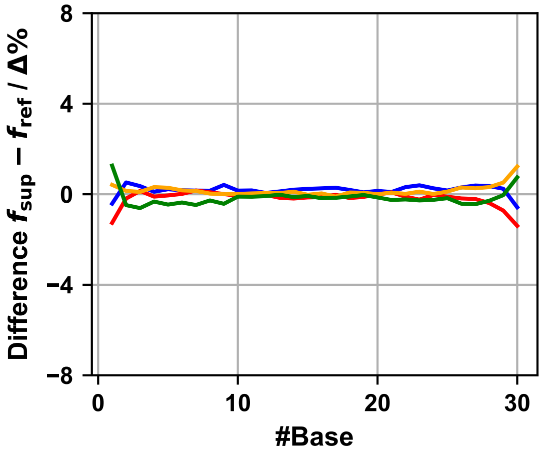

Fig. S6. Difference in nucleotide content between Supernatant and Reference. *Plotted is the nucleotide content per position of the Supernatant, subtracted by the nucleotide content per position of the Reference sample. The colours stand for the different bases, red being Adenine, blue Guanine, green Thymine and yellow Cytosine.*

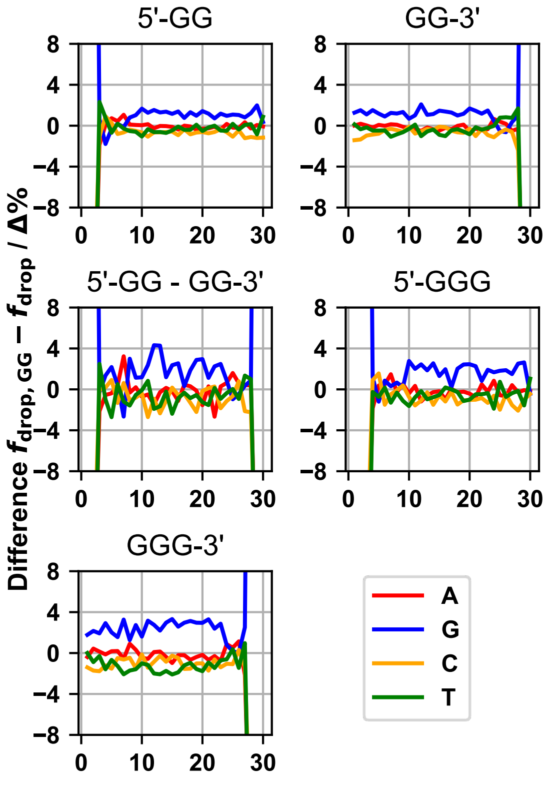

Fig. S7. Cooperative effect of multiple Gs at the terminus of sequences. *Sequences containing two or three consecutive G at the 5' or 3' terminus of a sequence have a higher probability of additional Gs in the same sequence. Plotted is the nucleotide content per position of sequences containing the defined number of Gs at the defined terminus in the droplet sample, subtracted by the nucleotide content of all sequences in the droplet sample. The colours stand for the different bases, red being Adenine, blue Guanine, green Thymine and yellow Cytosine.*

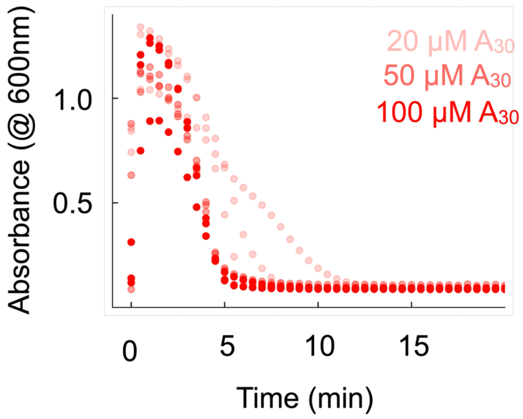

Fig. S8. Turbidity traces in the presence of different concentrations of A_{30} DNA. *Droplets were prepared according to standard conditions with 20 (light pink), 50 (red), or 100 µM(dark red) of A_{30}. Turbidity was observed as Absorbance at 600 nm on the UV VIS spectrometer. Lifetime is determined at a threshold of 0.12. Three independent experiments were performed for each condition (N=3). Source data is available in the source data file.*

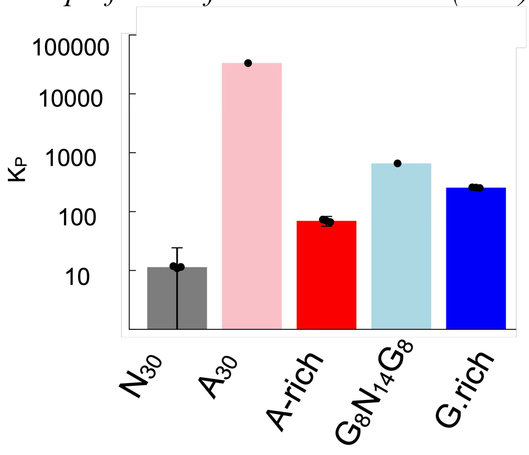

Fig. S9. Partitioning of A-rich and G-rich randomers compared to designed sequences. *DNA partitioning into the droplet phase was determined by preparing static droplets according to standard static droplet preparation, spinning down the samples, and measuring the DNA concentration in the supernatant using HPLC. The partition coefficient was then determined with the droplet volume for static droplets on the confocal microscope. Data is presented on a logarithmic scale. Error bars represent the error calculated from the standard deviation of the droplet volume determination and the three measurements of the DNA concentration in the supernatant. The shown datapoints are based on the three replicates of the supernatant concentrations (N=3). Source data is available in the source data file.*

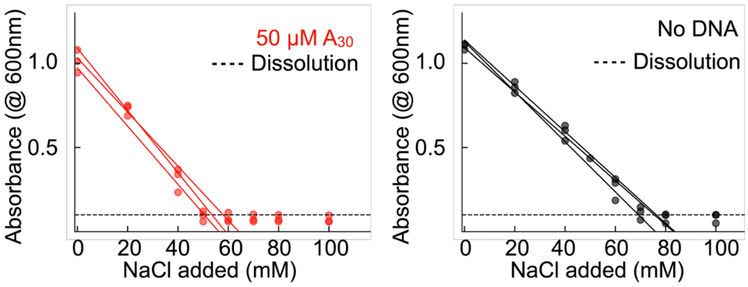

	Control	*A30*
CSC rep.1	*75.5*	*57.9*
CSC rep.2	*69.2*	*50.5*
CSC rep.3	*76.3*	*53.5*
CSC average	*73.7*	*54.0*
CSC standard deviation	*3.91*	*3.76*

Fig. S10. Critical salt concentration *Titration of static coacervate samples prepared according to standard static droplet protocol with 50 µM A30 (red) and without (black). 20 µL samples were titrated with concentrated NaCl solution, and the absorbance at 600 nm was measured on the UV VIS spectrometer after salt addition and mixing. The titrations were performed in triplicate(N=3). and each replicate was fit linearly with all datapoints above 0.12, as datapoints below no longer followed the linear trend during dissolution. The critical salt concentrations were determined at the intersection of the extrapolated fits with y=0.1. Source data is available in the source data file.*

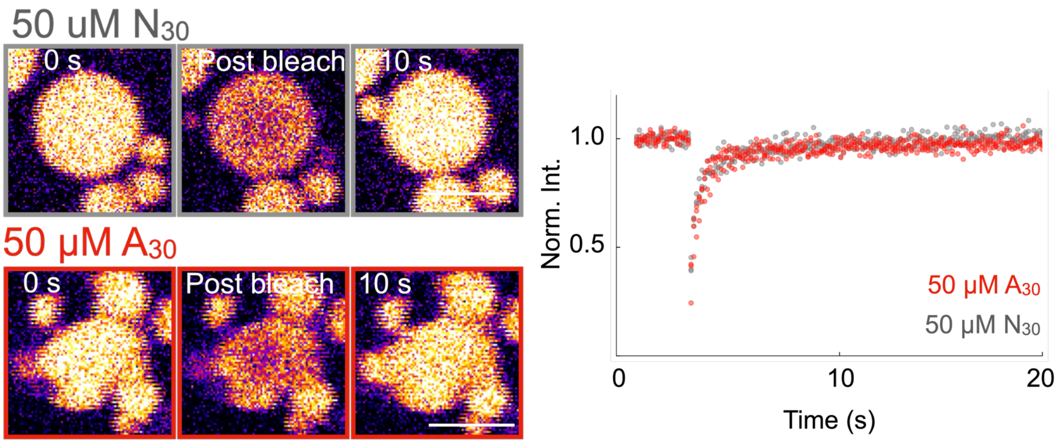

Figure S11: **Comparison of the precursor peptide diffusivities with 50 µM N_{30} or A_{30} DNA.** *Fluorescence recovery after photobleaching of fluorescently tagged precursor peptide NBD-G(RG)$_3$D-OH,(**A**) used at 1 mM concentration. Sample conditions were 20 mM precursor, 3 mM pseudo anhydride. 4.1 mM pU with 50 µM N_{30} (grey) or A_{30} (red). The photobleached peptide was observed with the confocal microscope for 70 s in total. Scalebars represent 10 µm. . Three independent measurements were performed for each condition (N=3). Source data is available in the source data file.*

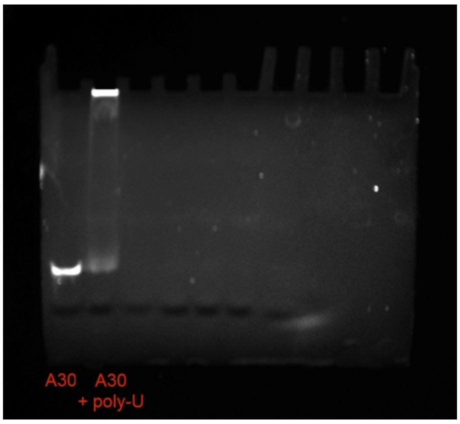

Fig. S12. Non-Denaturing gel electrophoresis of A$_{30}$ in the presence of poly-U. Non-denaturing 14% acrylamide gel was pre-run for 45 min then run for 1h10min at 180V in 1x TBE buffer in an ice bath. Cy-3 tagged A30 was run once without and once with Poly-U, which was used in 5x excess. Bands were imaged by fluorescent excitation of the Cy-3 dye on a gel imager by BioRad.

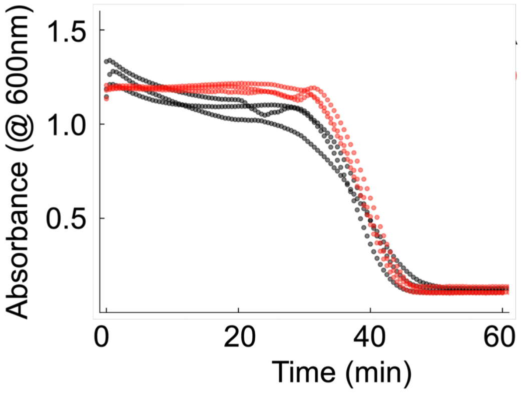

Fig. S13. Turbidity traces without DNA and in the presence of 50 µM A$_{30}$ using polystyrene sulfonate as alternative polyanion. *Droplets were prepared with 6 mM peptide, 2.5 mM polystyrene sulfonate 30 mM EDC without DNA (black) or with 50 µM of A$_{30}$* (red). *Absorbance measurements at 600nm were directly started after fuel addition on the UV VIS spectrometer. Three independent measurements were performed for each condition (N=3). Source data is available in the source data file.*

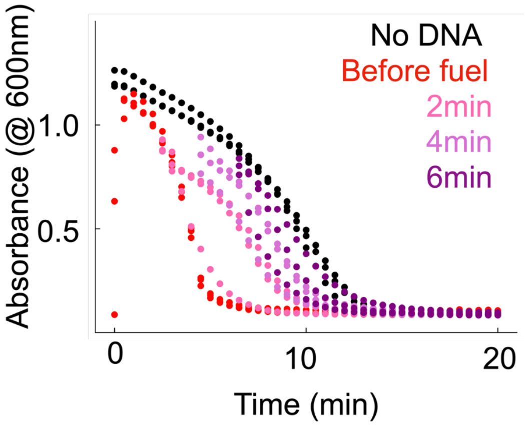

Fig. S14. Turbidity traces in the presence of 50 µM A_{30} added at different timepoints. *Droplets were prepared according to standard conditions with 50 µM of A_{30} added before fuel addition(red) (standard procedure), two(light pink), four (pink) or 6 minutes (purple) after fuel addition. Absorbance measurements at 600nm were directly started after A30 addition on the UV VIS spectrometer. Three independent measurements were performed for each condition (N=3). Source data is available in the source data file.*

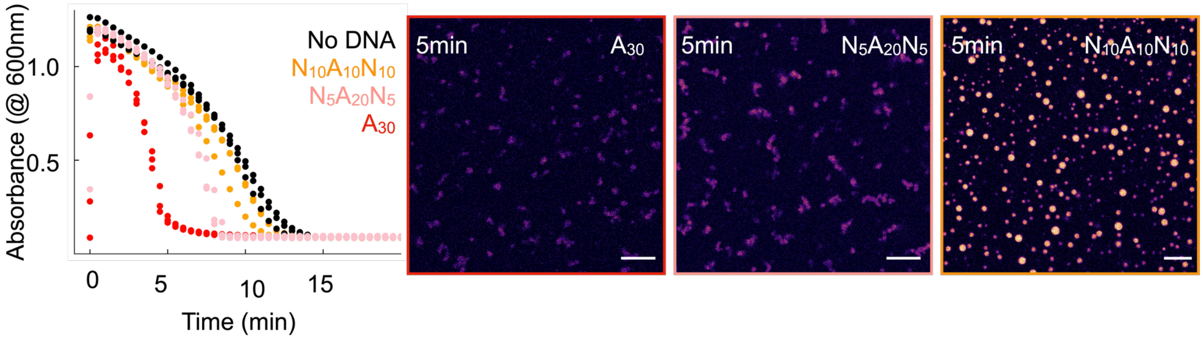

Figure S15: Droplet lifetime and morphology in the presence of central adenine stretches of increasing length. *Droplets were prepared according to standard conditions without (black) or with 50 µM of DNA: A30 (red), N10A10N10 (orange) or N5A20N5 (light pink) . Turbidity was observed as Absorbance at 600 nm on the UV VIS spectrometer. Lifetime is determined at a threshold of 0.12. Source data is available in the source data file. Three biological replicates were measured for each condition (N=3). Droplets were prepared according to standard conditions and imaged with 1 mM NBD-GRGRGRGD-OH on the glass after five minutes.Scalebars represent 10 µm. Micrographs are representative images of one biological replicate at the indicated timepoints. Three biological replicates were measured for each condition (N=3).*

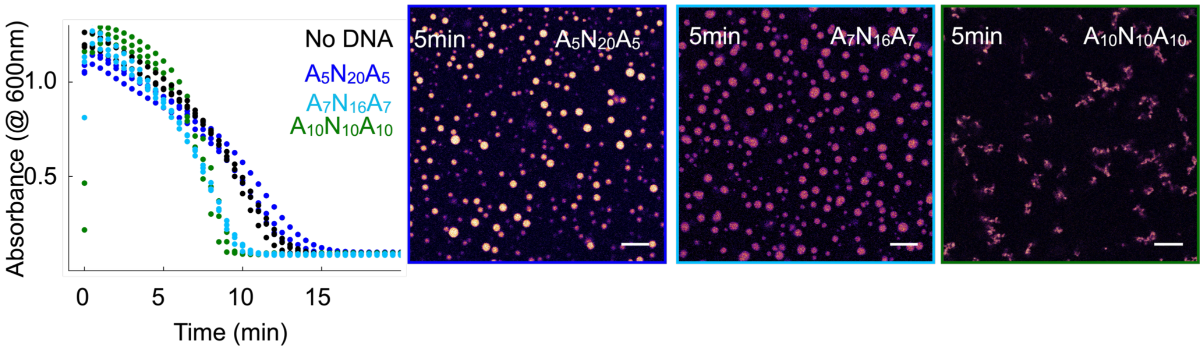

Figure S16: Droplet lifetime and morphology in the presence of terminal adenine stretches of increasing length. *Droplets were prepared according to standard conditions without (black) or with 50 µM of DNA: A5N20A5 (blue), A7N16A7 (light blue) or A10N10A10 (green) . Turbidity was observed as Absorbance at 600 nm on the UV VIS spectrometer. Lifetime is determined at a threshold of 0.12. Source data is available in the source data file.*

Three biological replicates were measured for each condition (N=3). Droplets were prepared according to standard conditions and imaged with 1 mM NBD-GRGRGRGD-OH on the glass after five minutes.Scalebars represent 10 µm. Micrographs are representative images of one biological replicate at the indicated timepoints. Three biological replicates were measured for each condition (N=3).

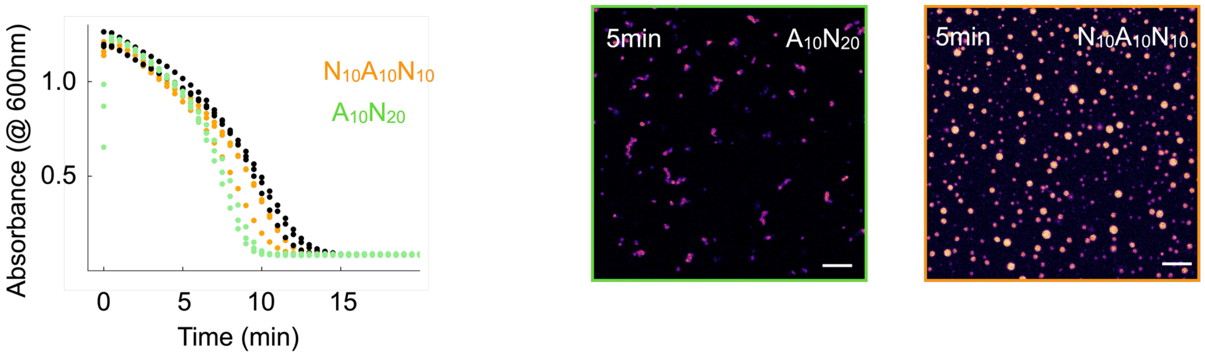

Figure S17. Droplet lifetime and morphology in the presence of central ten adenines compared to terminal ten adenines. *Droplets were prepared according to standard conditions without (black) or with 50 µM of DNA: N10A10N10 (orange) or NA10N20 (light green). Turbidity was observed as Absorbance at 600 nm on the UV VIS spectrometer. Lifetime is determined at a threshold of 0.12. Source data is available in the source data file. Droplets were prepared according to standard conditions and imaged with 1 mM NBD-GRGRGRGD-OH on the glass after five minutes. Scalebars represent 10 µm. Micrographs are representative images of one biological replicate at the indicated timepoints. Three biological replicates were measured for each condition (N=3).*

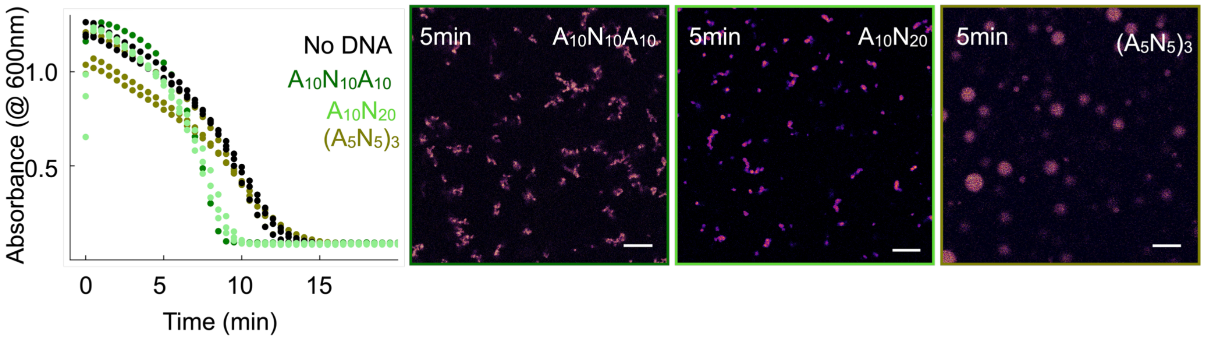

Figure S18. Droplet lifetime and morphology in the presence of differently distributed consecutive adenines. *We compare ten terminal adenines on one side to ten terminal adenines on both sides and fifteen distributed adenines in stretches of five.*
Droplets were prepared according to standard conditions without (black) or with 50 µM of DNA: A10N10A10 (dark green), A10N20 (light green) or (A5N5)3 (brown). Turbidity was observed as Absorbance at 600 nm on the UV VIS spectrometer. Lifetime is determined at a threshold of 0.12. Source data is available in the source data file. Three biological replicates were measured for each condition (N=3). Droplets were prepared according to standard conditions and imaged with 1 mM NBD-GRGRGRGD-OH on the glass after five minutes.

Scalebars represent 10 µm. Micrographs are representative images of one biological replicate at the indicated timepoints. Three biological replicates were measured for each condition (N=3).

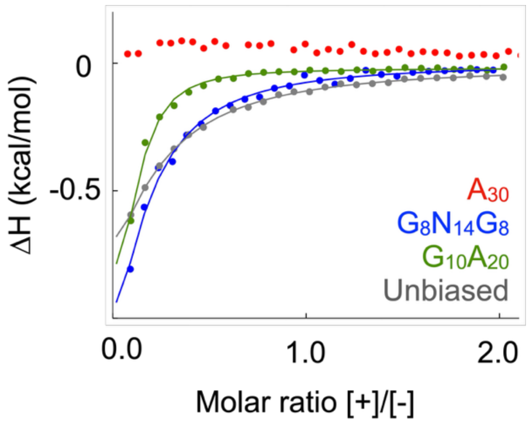

Sequence	Dissociation constant with AcF(RG)$_3$N-NH$_2$ (µM)	ΔH (kcal/mol)
N_{30}	188 ± 44	-3.7 ± 0.7
$G_8N_{14}G_8$	113 ± 32	-3.5 ± 1.3
$G_{10}A_{20}$	45 ± 13	-2.0 ± 0.5
A_{30}	No binding	0.03± 0.04

Fig. S19. ITC measurements of peptide model with different DNA 30mers. ITC titrations of 500 µM of different DNA 30mers (charge concentration) with 5 mM of the peptide anhydride model peptide model (AcF(RG)$_3$N-NH$_2$) (charge concentration). Three independent measurements are performed for each condition *(N=3)*, one replicate is shown here: A30 in red, G8N14G8 in blue, G10A20 in green and the N30 unbiased library in grey. Source data can be found in the source data file.

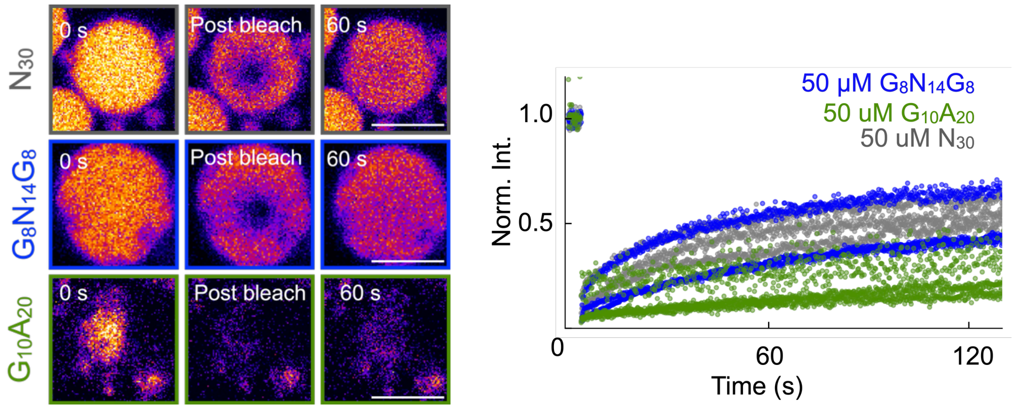

Fig. S20*:* Comparison of poly-U diffusivities with and without different G-rich DNA. *Fluorescence recovery after photobleaching of Cy5-A_{15} RNA oligomer hybridized to poly-U used at 500 nM concentration. Intensity is normalized to an averaged pre-bleach value (Norm. Int.) Grey datapoints represent the unbiased library N30, blue G8N14G8 and green G10A20. Sample conditions were 20 mM precursor, 3 mM pseudo anhydride, 4.1 mM poly-U with 50 µM $G_8N_{14}G_8$ or $G_{10}A_{20}$. The samples were observed for at least 120 s. Three independent samples (N=3) were measured for each condition. Scalebars represent 10 µm. Source data for the plots is available in the source data file.*

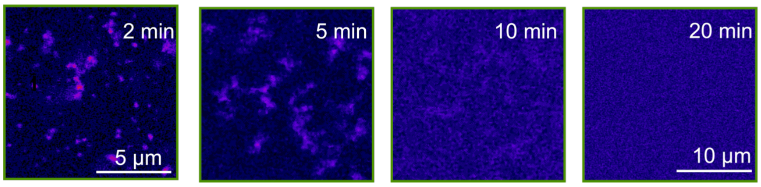

Fig. S21. Confocal micrographs of droplet formation in the presence of 50 µM $G_{10}A_{20}$. *Samples were prepared according to standard protocol, stained with 500 nM Sulforhodamine and observed under the confocal microscope on the glass, using an IBIDI chamber coated with a 5 %PVA solution. Micrographs are representative images of one biological replicate at the indicated timepoints. Three biological replicates were measured in total.*

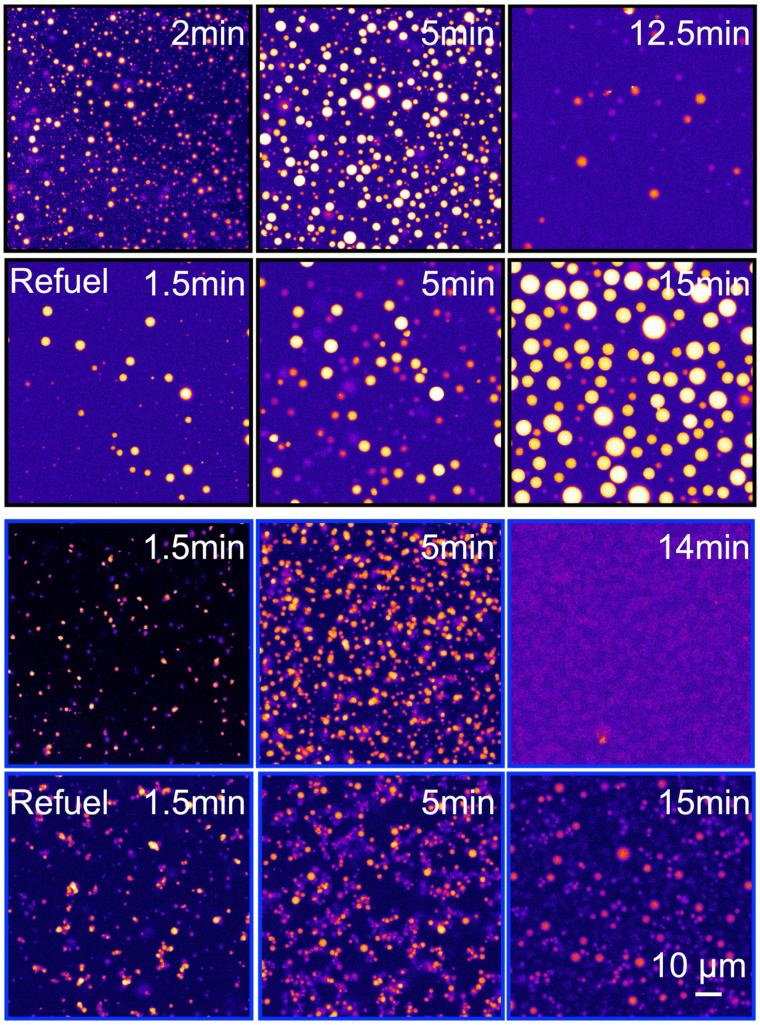

Fig. S22. Confocal micrographs of re-fueling experiments without DNA and with 50 µM $G_8N_{14}G_8$. *Samples were prepared according to standard protocol, stained with 500 nM Sulforhodamine and observed under the confocal microscope on the glass, using an IBIDI chamber coated with a 5 %PVA solution. Samples were refueled with 30 mM EDC shortly before droplet dissolution (12.5min.) for the no-DNA control, after 15 min. for the kinetically trapped $G_8N_{14}G_8$ sample. Micrographs are representative images of one biological replicate at indicated timepoints. Three biological replicates were measured for each condition (N=3).*

A) commercial

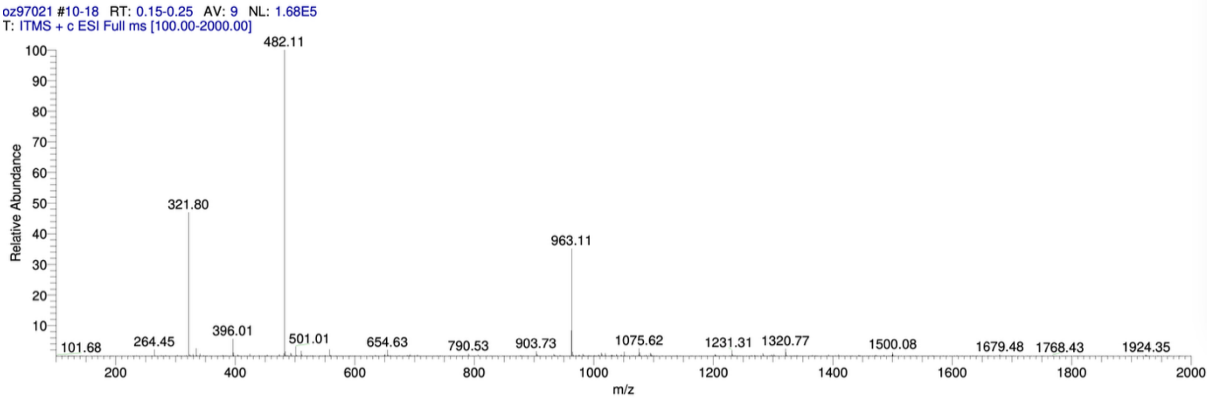

B) self synthesized

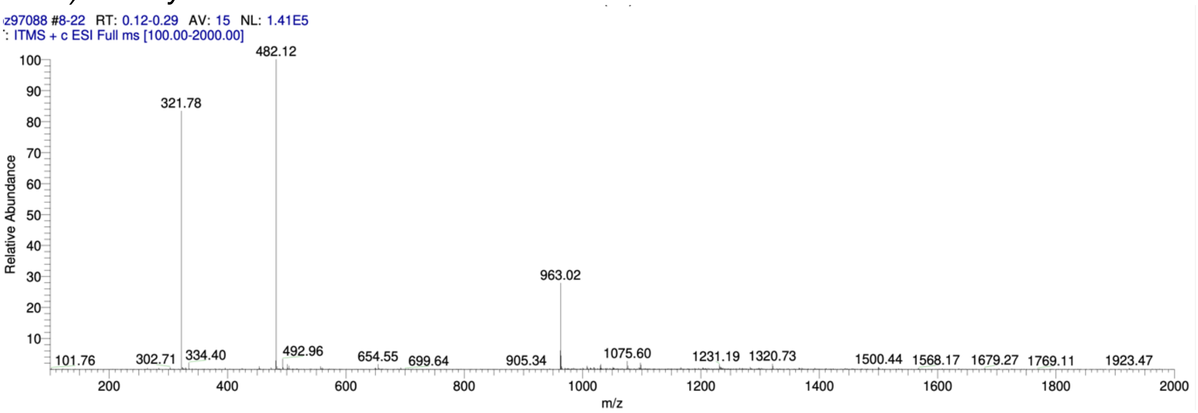

C) commercial

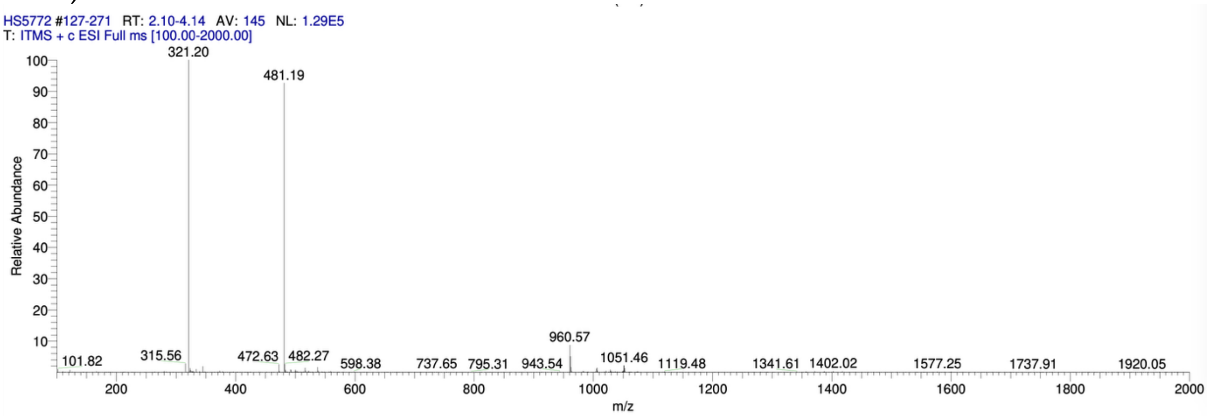

Supplementary Spectra: *Mass-Spectra from ESI-MS, Characterization of AcF(RG)3D-OH (A.commercial, B.self-synthesized) and AcF(RG)3N-NH2 (C. commercial.) The molecular weight of AcF(RG)3D-OH is 962.05, observed masses are 963.11 $(Mw+H)^+$ for the commercial and 963.02 $(Mw+H)^+$ for the self synthesized peptide. Also observed are the double $(Mw+2H)^{2+}$ and triple charged $(Mw+3H)^{3+}$ masses at 482.12 and 482.11 as well as 321.80 and 321.78 . The molecular weight of AcF(RG)3N-NH2 is 960.08, the observed mass is 960.57 $(Mw+H)^+$. Also observed are the double $(Mw+2H)^{2+}$ and triple charged $(Mw+3H)^{3+}$ masses at 481.19 and 321.20. For more detailed characterization see Supplementary table 8.*
