# Supplementary material for: DNA affects the phenotype of fuel-dependent coacervate droplets
Source: Nat Commun. 2026 Mar 26;17:2953. doi: 10.1038/s41467-026-71024-8 (PMC13031873; doi:10.1038/s41467-026-71024-8)
Supplement: Supplementary file 2 — Description of Additional Supplementary Files [file 41467_2026_71024_MOESM2_ESM.pdf]

Description of Additional Supplementary Files

Supplementary Movie 1: Lifecycle of Droplets without added DNA under standard conditions stained with 500nM Sulforhodamine.

Supplementary Movie 2: Shortened Lifecycle of Droplets with added 50 µM A30 DNA stained with 500nM Sulforhodamine.
