# Supplementary material for: DNA affects the phenotype of fuel-dependent coacervate droplets
Source: Nat Commun. 2026 Mar 26;17:2953. doi: 10.1038/s41467-026-71024-8 (PMC13031873; doi:10.1038/s41467-026-71024-8)
Supplement: Supplementary file 5 — Reporting Summary [file 41467_2026_71024_MOESM5_ESM.pdf]

nature portfolio

Corresponding author(s): Job Boekhoven

Last updated by author(s): Feb 20, 2026

Reporting Summary

Nature Portfolio wishes to improve the reproducibility of the work that we publish. This form provides structure for consistency and transparency in reporting. For further information on Nature Portfolio policies, see our Editorial Policies and the Editorial Policy Checklist.

Statistics

For all statistical analyses, confirm that the following items are present in the figure legend, table legend, main text, or Methods section.

n/a	Confirmed	
☐	☒	The exact sample size (*n*) for each experimental group/condition, given as a discrete number and unit of measurement
☐	☒	A statement on whether measurements were taken from distinct samples or whether the same sample was measured repeatedly
☐	☒	The statistical test(s) used AND whether they are one- or two-sided<br>*Only common tests should be described solely by name; describe more complex techniques in the Methods section.*
☒	☐	A description of all covariates tested
☒	☐	A description of any assumptions or corrections, such as tests of normality and adjustment for multiple comparisons
☐	☒	A full description of the statistical parameters including central tendency (e.g. means) or other basic estimates (e.g. regression coefficient) AND variation (e.g. standard deviation) or associated estimates of uncertainty (e.g. confidence intervals)
☐	☒	For null hypothesis testing, the test statistic (e.g. *F*, *t*, *r*) with confidence intervals, effect sizes, degrees of freedom and *P* value noted<br>*Give P values as exact values whenever suitable.*
☒	☐	For Bayesian analysis, information on the choice of priors and Markov chain Monte Carlo settings
☒	☐	For hierarchical and complex designs, identification of the appropriate level for tests and full reporting of outcomes
☒	☐	Estimates of effect sizes (e.g. Cohen's *d*, Pearson's *r*), indicating how they were calculated

Our web collection on statistics for biologists contains articles on many of the points above.

Software and code

Policy information about availability of computer code

Data collection	The numerical generator can be found on Github at https://doi.org/10.5281/zenodo.18597396.To collect our data on the confocal we used the Leica LAS X software Version 4.6, on the UV spectrometer we used the SkanIt Software Version 6.0, on the HPLC we used Chromeleon Version 7.3.2, on the ITC we used MicroCal PEAQ-ITC Control software(2022).
Data analysis	We have used FIJI (ImageJ), Python(3.13), Chromeleon Version 7.3.2 and Microsoft Excel to analyze our data. The FRAP analysis script and scripts to generate the final data and figures from raw sequencing data can be found on Github at https://doi.org/10.5281/zenodo.18597396.

For manuscripts utilizing custom algorithms or software that are central to the research but not yet described in published literature, software must be made available to editors and reviewers. We strongly encourage code deposition in a community repository (e.g. GitHub). See the Nature Portfolio guidelines for submitting code & software for further information.

Data

Policy information about availability of data

All manuscripts must include a data availability statement. This statement should provide the following information, where applicable:

- Accession codes, unique identifiers, or web links for publicly available datasets
- A description of any restrictions on data availability
- For clinical datasets or third party data, please ensure that the statement adheres to our policy

The raw sequencing data generated in this study have been deposited in the Sequence Read Archive (SRA) of the National Center for Biotechnology Information

(NCBI): https://www.ncbi.nlm.nih.gov/sra/PRJNA1424544. The other experimental data generated in this study are provided in the Supplementary Information/ Source Data file. Source images are available upon request to Job Boekhoven due to their size, permanent access can be given with one month response time.

Research involving human participants, their data, or biological material

Policy information about studies with human participants or human data. See also policy information about sex, gender (identity/presentation), and sexual orientation and race, ethnicity and racism.

Reporting on sex and gender	not applicable as no humans are involved
Reporting on race, ethnicity, or other socially relevant groupings	not applicable as no humans are involved
Population characteristics	not applicable as no humans are involved
Recruitment	not applicable as no humans are involved
Ethics oversight	not applicable as no humans are involved

Note that full information on the approval of the study protocol must also be provided in the manuscript.

Field-specific reporting

Please select the one below that is the best fit for your research. If you are not sure, read the appropriate sections before making your selection.

☒ Life sciences ☐ Behavioural & social sciences ☐ Ecological, evolutionary & environmental sciences

For a reference copy of the document with all sections, see nature.com/documents/nr-reporting-summary-flat.pdf

Life sciences study design

All studies must disclose on these points even when the disclosure is negative.

Sample size	No sample size calculation was done. Typical number of replicates was three, due to cost benefits considerations.
Data exclusions	No data was excluded.
Replication	Independent replicates were performed. No issues of reproducibility were encountered.
Randomization	This does not apply to our study either. Both the control samples and the different samples we compare are made up from the same stock solutions, only the composition changes/additional components are added.
Blinding	No blinding was done as data collection and analysis was done by the same person. It is not really applicable to our field.

Reporting for specific materials, systems and methods

We require information from authors about some types of materials, experimental systems and methods used in many studies. Here, indicate whether each material, system or method listed is relevant to your study. If you are not sure if a list item applies to your research, read the appropriate section before selecting a response.

Materials & experimental systems

n/a	Involved in the study	
☒	☐	Antibodies
☒	☐	Eukaryotic cell lines
☒	☐	Palaeontology and archaeology
☒	☐	Animals and other organisms
☒	☐	Clinical data
☒	☐	Dual use research of concern
☒	☐	Plants

Methods

n/a	Involved in the study	
☒	☐	ChIP-seq
☒	☐	Flow cytometry
☒	☐	MRI-based neuroimaging

Plants

Seed stocks	no plants were used
Novel plant genotypes	no plants were used
Authentication	no plants were used
